# Probing Oral Microbial Functionality – Expression of *spxB* in Plaque Samples

**DOI:** 10.1371/journal.pone.0086685

**Published:** 2014-01-29

**Authors:** Lin Zhu, Yifan Xu, Joseph J. Ferretti, Jens Kreth

**Affiliations:** 1 Department of Periodontics, College of Dentistry, University of Oklahoma Health Sciences Center, Oklahoma City, Oklahoma, United States of America; 2 Department of Microbiology and Immunology, College of Medicine, University of Oklahoma Health Sciences Center, Oklahoma City, Oklahoma, United States of America; 3 Department of Surgical Oncology, The First Hospital of China Medical University, Shenyang, People's Republic of China; 4 Department of Oral Biology, College of Dentistry, University of Oklahoma Health Sciences Center, Oklahoma City, Oklahoma, United States of America; Virginia Commonwealth University, United States of America

## Abstract

The Human Oral Microbiome Database (HOMD) provides an extensive collection of genome sequences from oral bacteria. The sequence information is a static snapshot of the microbial potential of the so far sequenced species. A major challenge is to connect the microbial potential encoded in the metagenome to an actual function in the *in vivo* oral biofilm. In the present study we took a reductionist approach and identified a considerably conserved metabolic gene, *spxB* to be encoded by a majority of oral streptococci using the HOMD metagenome information. *spxB* encodes the pyruvate oxidase responsible for the production of growth inhibiting amounts of hydrogen peroxide (H_2_O_2_) and has previously been shown as important in the interspecies competition in the oral biofilm. Here we demonstrate a strong correlation of H_2_O_2_ production and the presence of the *spxB* gene in dental plaque. Using Real-Time RT PCR we show that *spxB* is expressed in freshly isolated human plaque samples from several donors and that the expression is relative constant when followed over time in one individual. This is the first demonstration of an oral community encoded gene expressed *in vivo* suggesting a functional role of *spxB* in oral biofilm physiology. This also demonstrates a possible strategy to connect the microbial potential of the metagenome to its functionality in future studies by identifying similar highly conserved genes in the oral microbial community.

## Introduction

The Human Oral Microbiome Database (HOMD) contains DNA sequence information from over 1300 genomes [1]. This number is most likely to grow in the future. The wealth of information provided allows the interested researcher to screen the available sequence data for common features and investigate a biological relevant relation to the community structure. For example is a specific gene or a set of genes associated with a healthy community or a predictor of a pathogenic oral biofilm?

One caveat of this approach is that the metagenome only gives a static snapshot of the oral bacterial community potential at the time of sampling [2]. It will not allow determining a dynamic relationship or the functionality of the bacterial community at any given time. While the potential is determined by the metagenome, the functionality of the bacterial community is driven by its metatranscriptome and metaproteome, thus at the RNA and protein level [2], [3]. However, investigating the metatranscriptome and the metaproteome comes with certain challenges, including the amount of RNA or protein required to successfully cover the entire metatranscriptome or metaproteome of any given plaque sample. For example detection and quantification of low abundance transcripts by RNA Seq can be challenging [4] since the starting material for RNA isolation, dental plaque, is limited. Furthermore, the amount of species open reading frames would lead to large amounts of diverse sequences reads requiring specific software to adequately manage the data [5]–[7].

Alternatively, the metagenomic information could be used to identify an ecological relevant oral community encoded gene. The expression of this gene in human plaque samples could be determined directly thus probing its functionality *in vivo*. Ideally several members of the oral microbial biofilm share this gene. We previously identified a high homology of the pyruvate oxidase gene *spxB* between the oral commensal *Streptococcus sanguinis* and *Streptococcus gordonii* [8]. SpxB is an oxido-reductase, catalyzing the conversion of pyruvate to acetyl phosphate, CO_2_ and H_2_O_2_ under aerobic conditions [9], [10]. The gene provides several advantages for the encoding species. Initially, we identified H_2_O_2_ as inhibiting substance in the dual species competition with cariogenic *Streptococcus mutans* [11], [12]. Deletion of the *spxB* gene in both *S. sanguinis* and *S. gordonii* rendered them non-competitive against *S. mutans* demonstrating that SpxB is responsible for competitive H_2_O_2_ production [12]. Besides the inhibitory H_2_O_2_ action, two additional effects increasing commensals competitiveness are obvious: i) ATP production from acetyl phosphate for energy generation and ii) H_2_O_2_ induced release of extracellular DNA (eDNA) [12]. eDNA is a major component of the extracellular polymeric substance (EPS) of biofilms, promoting cell-cell and cell-tooth contact [13], [14]. H_2_O_2_ induced release of eDNA can also serve in horizontal gene transfer promoting genetic diversity as we demonstrated recently [15]. In addition, the ability to produce inhibitory amounts of H_2_O_2_ seems to be limited to oral streptococci [16].

In this pilot study, we demonstrate that *spxB* is a suitable candidate gene encoded by several important commensal streptococci, identified by homologous sequence search using the HOMD database. Oral streptococcal production of H_2_O_2_ as measured with specific indicator plates seems to correlate with the presence of the *spxB* gene as determined with PCR and *spxB* specific oligonucleotides. Furthermore, we isolated RNA from freshly isolated human plaque samples and demonstrate that *spxB* is expressed with Real-Time RT-PCR, suggesting a functional role in the oral biofilm.

## Materials and Methods

### Ethics Statement

The Institutional Review Board of University of Oklahoma HSC approved the study protocol for human subjects. IRB protocol # 1934. Participants signed a written consent form following an approved procedure by the IRB.

### Bacterial Strains and Growth Conditions

Bacterial strains are listed in Tab. 1. Bacteria were routinely grown aerobically in 5% CO_2_ at 37°C overnight in BHI medium (Brain Heart Infusion; Difco, Sparks, MD) or on BHI plates, or as otherwise indicated.

**Table 1:** Bacterial strains.

Strain	Reference
***S. mutans*** ** UA140**	[31]
***S. mutans*** ** UA159**	[32]
***S. mitis*** ** 12261**	[33]
***S. gordonii*** ** DL1**	[34]
***S. gordonii*** ** V288**	[35]
***S. oralis*** ** MC3-1**	[36]
***S. oralis*** ** J22**	[36]
***S. sanguinis*** ** SK36**	[37]
***S. sanguinis*** ** 133–79**	[38]
***S. salivarius***	Clinical isolates
***S. infantis***	
***S. parasanguinis***	
***S. mutans*** ** serotype F**	
***S. mutans*** ** serotype K**	
***S. gordonii***	

### Subjects and Plaque Sampling

The present study sole intention was to determine the feasibility of measuring *spxB* gene expression in dental plaque samples. Therefore, no subject related data were collected from the 8 volunteers asked to donate plaque samples. From each individual, supragingival dental plaque samples were recovered between 16 and 18 hours after tooth brushing, from interproximal, vestibular and lingual surfaces of all teeth using a dental probe. The collected plaque samples were immediately removed from the dental probe after a visible amount has built up using a sterile tip and resuspended in 1 ml TRIzol (lifetechnologies). The sampling procedure lasted not longer than five minutes. Resuspended plaque samples in TRIzol were immediately frozen at −80°C until further processing the next day. Plaque samples for plating were removed from the teeth using a cotton swap and directly plated onto the H_2_O_2_ indicator plates.

### RNA Isolation and cDNA Synthesis

To isolate RNA, cells were disrupted three times for 30 seconds each using 0.1 mm zirconia/silica beads (BioSpec, Inc) in a FastPrep FP210 Homogenizer (Thermo Scientific) using the highest speed setting. Total RNA isolation from TRIzol was carried out according to the manufacturer’s instructions (Isolation of total RNA using TRIzol, lifetechnologies). RNA samples were treated with Turbo DNase to remove traces of chromosomal DNA after manufacturers recommendations (Ambion). RNeasy MiniElute cleanup kit (Qiagen) was used to purify RNA samples after DNase treatment. 2 µl RNA was immediately run on a 1% agarose gel to check for integrity. cDNA was synthesized using qScript™ cDNA synthesis kit (Quanta Biosciences) according to the manufacturer’s protocol.

### RNA Integrity

RNA integrity was determined using an Agilent 2100 Bioanalyzer (Agilent Technologies, Inc.). The yield of the extracted RNA and intactness of rRNA was assayed from 1 µl RNA after DNase treatment and RNeasy MiniElute cleanup in a 2100 BioAnalyzer using the protocol of the RNA 6000 Nano Lab Chip Kit (Agilent Technologies, Inc.). The chip allows for analyzing 12 samples simultaneously. RNA was therefore collected first and stored at −80°C until all 8 samples were available for the RNA 6000 Nano Lab Chip run.

### RT-PCR and Real-Time PCR

RT-PCR was performed as described earlier [17]. Real-time RT PCR was performed to determine specific cDNA copies with the comparative threshold cycle (CT) method using a MyiQ single-color real-time PCR detection system (Bio-Rad) and PerfeCtaTM SYBR^®^ Green SuperMix for iQ™ (Quanta Biosciences). Relative changes in cDNA copies representing differential gene expression were calculated using the ΔC_T_ method described previously [18]. The 16S rRNA gene was used as the housekeeping reference gene. Oligonucleotides were synthesized by IDTDNA. Oligonucleotide sequences are: 16S rRNA F - 5′-AAGCAACGCGAAGAACCTTA-3′; 16S rRNA R - 5′-GTCTCGCTAGAGTGCCCAAC-3′; universal spxB F - 5′-CATCATGGGTGACGGTGCAT-3′; universal spxB R - 5′-GCGTTAGGGAAGTCACAACC-3′.

### PCR

Colony PCR was performed by scraping a small amount of cells from the respective agar plate using a sterile pipet-tip and resuspending the cells in a pre-aliquoted PCR reaction mix. Alternatively, cells were inoculated in 2 ml BHI overnight; 1 ml was transferred into tubes containing lysing matrix B (MP Biomedicals, Solon, OH) and cells were homogenized in a FastPrep FP210 homogenizer (Thermo Scientific) (speed setting of 6.5). After centrifugation for 10 min at 13.200 rpm in a tabletop centrifuge, 2 µl supernatant containing chromosomal DNA was used as PCR template. PCR was performed with a G-Storm GS1 thermocycler (Gene Technologies) according to the manufacturer’s protocol. GoTaq-DNA polymerase was obtained from Promega, and oligonucleotides specific for *spxB* and 16S rRNA were the same as listed above.

### Detection of H_2_O_2_ Production

Indicator plates for H_2_O_2_ production were prepared and used as described [19]. This indicator plates allow for the detection of bacterial H_2_O_2_ production resulting in a blue pigment (Prussian blue, ferric ferrocyanide) that forms in the presence of H_2_O_2_. Plates were aerobically cultured in 5% CO_2_ at 37°C overnight.

## Results

### Prevalence of the *spxB* Gene in the Oral Biofilm Community

A BLAST search was performed using the *S. gordonii* CH1 nucleotide sequence as query against all available oral microbial genomes on the HOMD server. In addition to *S. gordonii*, the following oral *Streptococci* encode *spxB* homologs: *Streptococcus sanguinis*, *Streptococcus mitis*, *Streptococcus infantis*, *Streptococcus oralis*, *Streptococcus oligofermentans* and *Streptococcus cristatus* (Tab. 2). Furthermore, *Streptococcus pneumoniae* encodes also *spxB* as reported in the literature [20] and found during the BLAST search, but was omitted since it is usually not found in the dental associated oral biofilm. The homology of the *spxB* genes encoded by the oral streptococcal community is high ranging from 93% to 97% on the nucleotide level (against *S. gordonii* CH1) with a core sequence of about 1700 bp when compared to the 2308 bp of *S. gordonii* CH1. This suggests that the *spxB* gene is highly conserved among oral Streptococci. Interestingly, no homolog was found in cariogenic *Streptococcus mutans* confirming our previous observation that *S. mutans* does not produce competitive amounts of H_2_O_2_ [11].

**Table 2:** Distribution of *spxB* among oral streptococci.

Strain	Similarity
*Streptococcus gordonii* CH1	2308/2308 (100%)
*Streptococcus sanguinis* SK49	2000/2073 (96%)
*Streptococcus mitis* SK321	1978/2061 (95%)
*Streptococcus mitis* NCTC 12261	1975/2061 (95%)
*Streptococcus sanguinis* SK36	2061/2180 (94%)
*Streptococcus mitis* SK579	1969/2061 (95%)
*Streptococcus mitis* SK1080	1966/2061 (95%)
*Streptococcus sanguinis* SK72	2153/2309 (93%)
*Streptococcus sanguinis* SK115	1973/2071 (95%)
*Streptococcus infantis* X	1951/2061 (94%)
*Streptococcus oralis* SK255	1772/1824 (97%)
*Streptococcus sanguinis* SK340	1960/2073 (94%)
*Streptococcus sanguinis* ATCC 29667	1960/2073 (94%)
*Streptococcus sanguinis* ATCC 49296	1769/1824 (96%)
*Streptococcus oralis* SK1074	1768/1823 (96%)
*Streptococcus mitis* bv. 2 str. F0392	1768/1823 (96%)
*Streptococcus mitis* bv. 2 str. SK95	1763/1817 (97%)
*Streptococcus sanguinis* SK150	1953/2071 (94%)
*Streptococcus sanguinis* VMC66	2033/2180 (93%)
*Streptococcus oralis* SK304	1758/1817 (96%)
*Streptococcus sanguinis* SK160	1951/2073 (94%)
*Streptococcus oralis* Uo5	1751/1809 (96%)
*Streptococcus mitis* ATCC 6249	1742/1798 (96%)
*Streptococcus sanguinis* SK353	1948/2071 (94%)
*Streptococcus mitis* SK575	1754/1816 (96%)
*Streptococcus oralis* SK100	1740/1798 (96%)
*Streptococcus mitis* SK1073	1738/1798 (96%)
*Streptococcus oralis* ATCC 35037	1753/1823 (96%)
*Streptococcus mitis* SK564	1751/1822 (96%)
*Streptococcus infantis* SK1302	1742/1813 (96%)
*Streptococcus cristatus* ATCC 51100	1745/1817 (96%)
*Streptococcus mitis* B6	1728/1796 (96%)
*Streptococcus oligofermentans* AS 1.3089	1748/1824 (95%)
*Streptococcus infantis* ATCC 700779	1723/1797 (95%)
*Streptococcus oralis* SK610	1743/1824 (95%)
*Streptococcus mitis* SK616	1723/1798 (95%)

### Detection of *spxB* with *spxB* Universal Oligonucleotides

**Figure 1:**
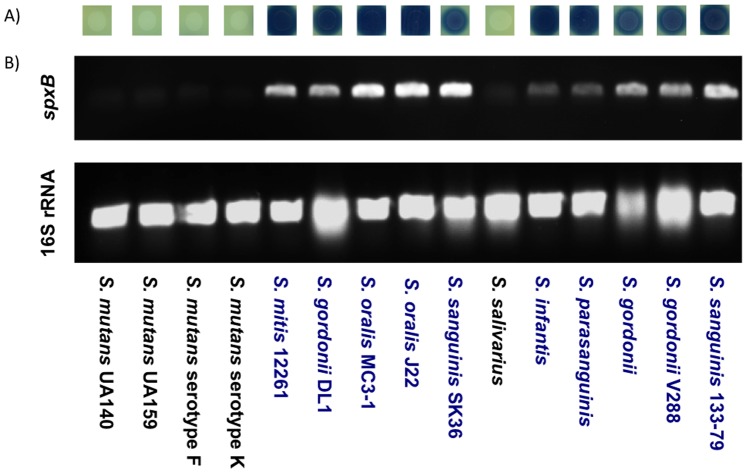
Correlation of H_2_O_2_ production and genomic *spxB* presence. **A) H_2_O_2_ production assay of common laboratory strains.** Cells were spotted on H_2_O_2_ indicator plates (Prussian Blue pigment development indicates H_2_O_2_ production) and incubated over night under aerobic conditions. Blue color development indicates H_2_O_2_ production. B) PCR using universal *spxB* oligonucleotides. Cells were scraped from the H_2_O_2_ indicator plates, grown overnight and chromosomal DNA isolated and used for PCR with universal *spxB* oligonucleotides. 16S rRNA amplification was performed as control for successful bacterial lysis during colony PCR.

The available sequence data for *spxB* from oral Streptococci was used to design an *spxB* specific set of oligonucleotides. Initially, the oligonucleotides were tested for their ability to amplify *spxB* from several *Streptococci* present in our laboratory culture collection. The H_2_O_2_ production potential of the here used streptococci was assessed first by inoculating 10 µl of an overnight culture on specific H_2_O_2_ indicator plates. The plates were incubated overnight to allow for growth and H_2_O_2_ production. As shown in Fig. 1 A, with the exception of *S. mutans* and *S. salivarius*, all other *Streptococci* produced H_2_O_2_ evident from the formation of a blue pigment. Subsequently, cells were removed and chromosomal DNA isolated to perform PCR with 16S rRNA and *spxB* specific oligonucleotides. The 16S rRNA control showed amplification for all strains tested. A clear correlation was evident between positive *spxB* amplification in *S. mitis*, *S. gordonii*, *S. oralis*, *S. sanguinis*, *S. infantis* and *S. parasanguinis* and the formation of the blue pigment (Fig. 1B). No significant amplification was observed for all *S. mutans* strains and *S. salivarius*, but we did recognize weak bands after longer integration during picture documentation (Fig. 1B).

**Figure 2:**
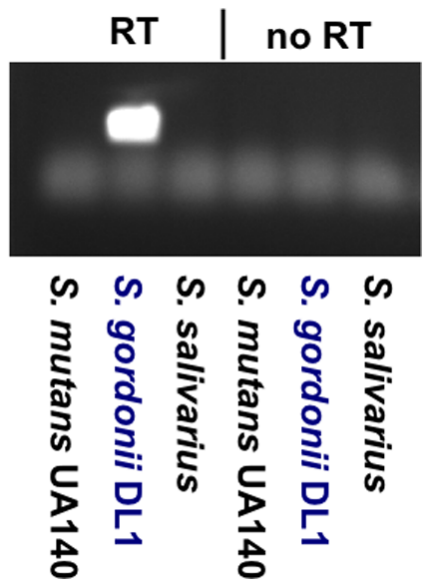
RT-PCR of cDNA from selected oral streptococci. RNA was isolated from the H_2_O_2_ producer *S. gordonii* DL1 and expression of *spxB* compared to H_2_O_2_-non-producer *S. mutans* UA140 and *S. salivarius*. RT*-*PCR was performed for 28 cycles.

To determine if the observed faint bands would interfere with our intention to measure *spxB* expression by giving false positive amplification products, RNA was isolated from *S. mutans*, *S. gordonii* and *S. salivarius* and cDNA synthesized. RT-PCR with *spxB* oligonucleotides showed only amplification for *S. gordonii* in the RT-PCR reaction, but no amplification for the no RT control (Fig. 2). 16S rRNA amplification was positive in all three RT-PCR reactions (data not presented). This suggests that the newly designed oligonucleotides are able to amplify *spxB* from H_2_O_2_ positive oral *Streptococci* and are suitable to be used in gene expression analysis.

### PCR Amplification of *spxB* from H_2_O_2_ Positive Plaque Colonies

**Figure 3:**
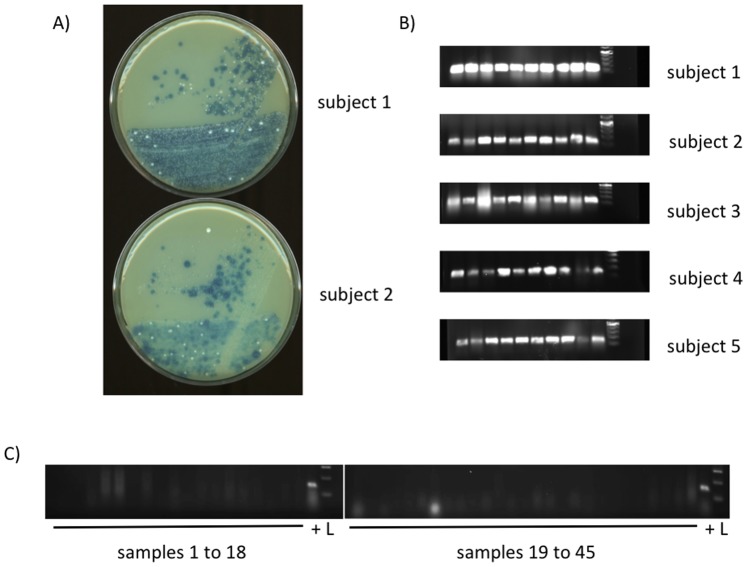
H_2_O_2_ production and *spxB* presence in plaque samples. A) Plaque samples for spreading on the H_2_O_2_ indicator plate were collected by swapping over several tooth with a sterile cotton swap. After inoculation, the indicator plates were incubated over night aerobically. Shown are representative plates from two subjects. B) Respective blue colonies indicating H_2_O_2_ production were picked and used for genomic *spxB* amplification. 16S rRNA was used as positive control, with bands for all tested colonies (not shown). C) 45 white colonies were also tested for spxB amplification. L = ladder,+ = chromosomal DNA *S. gordonii* DL1.

To further evaluate the *spxB* amplification potential of the newly designed *spxB* specific oligonucleotides, dental plaque was collected from five subjects. The plaque samples were inoculated on H_2_O_2_ indicator plates to separate single colonies. Fig. 3A represents plaque samples from two subjects and illustrates that colonies with and without blue pigment can be distinguished. Ten blue colonies from each subject were picked and used for colony PCR with the *spxB* specific oligonucleotides (Fig. 3B). PCR amplification was successful for all colonies in the five subjects. Obvious, however, was the difference in PCR efficiency. While subject 1 had strong bands for all ten colonies, subject 4 showed some heterogeneity in the band intensity (Fig. 3B). In addition, 45 white colonies were subject to PCR amplification with the *spxB* oligonucleotides. No amplification was observed (Fig. 3C). In summary, the *spxB* specific oligonucleotides are able to amplify *spxB* from freshly isolated H_2_O_2_ positive plaque bacteria suggesting that the oligonucleotides could be used in general to evaluate the presence of this gene in human plaque samples.

### RNA Isolation from Plaque Samples

**Figure 4:**
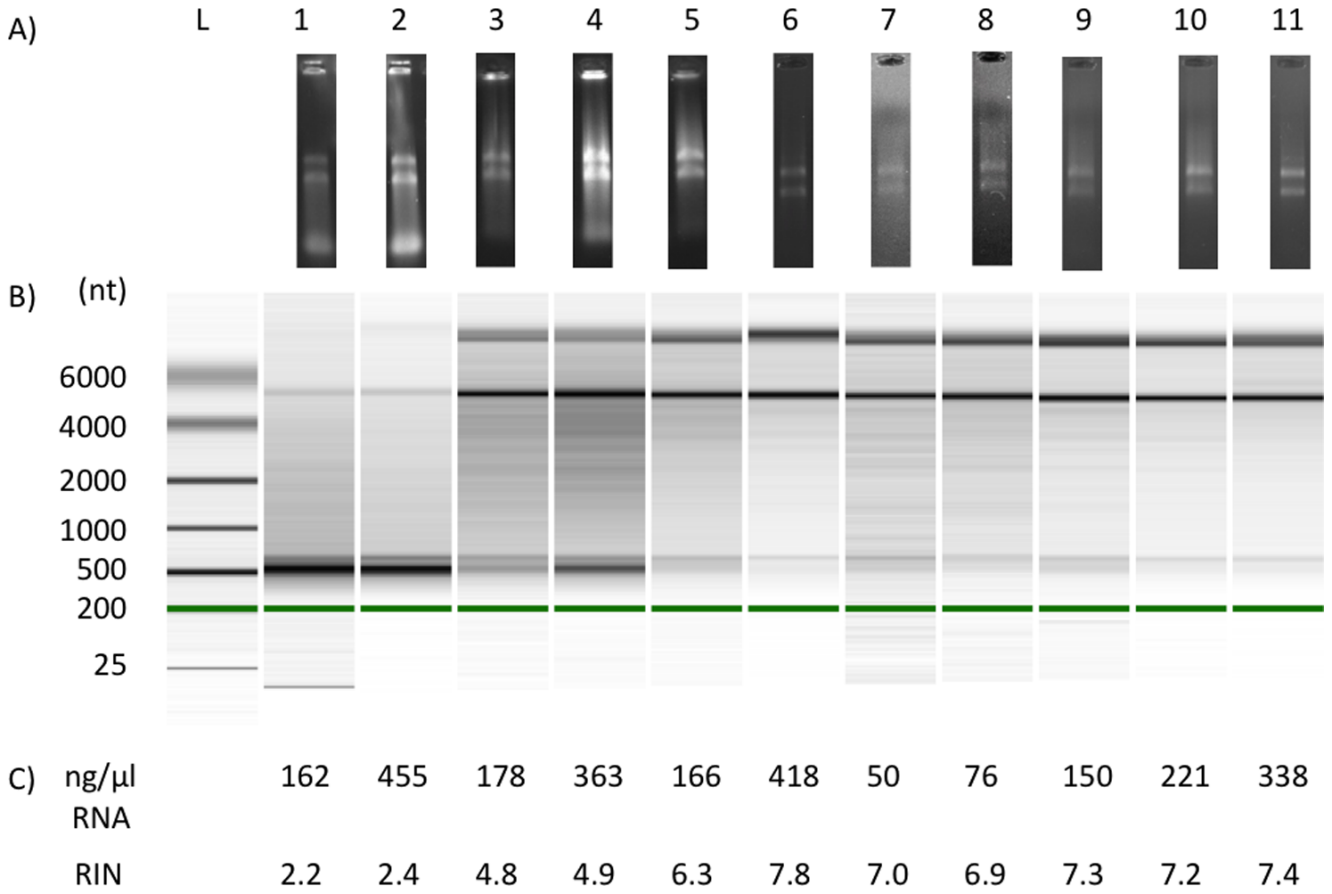
RNA integrity and concentration. A) Gel-eletrophoretic separation of isolated total RNA after DNase digest and clean-up on 1% agarose. B) Gel images of RNA samples generated by the Agilent Bioanalyzer using RNA 6000 Nano Lab Chip. C) RNA concentration and RIN as determined by the Agilent Bioanalyzer. RIN = RNA Integrity Number; L = RNA Ladder. Green line in Figure 4B: Bioanalyzer internal marker.

The goal of this study is to detect the expression of *spxB* from freshly isolated dental plaque samples to evaluate the *in vivo* expression of this gene. The major challenge in the detection of gene expression from host-derived biofilms is the isolation of sufficient amounts of high quality RNA [21]. To test feasibility of RNA isolation, plaque was sampled from 11 subjects asked to refrain from tooth brushing in the morning to collect a sufficient amount of plaque in the afternoon. The plaque samples were immediately processed for RNA isolation. Isolated RNA was analyzed and RIN (RNA Integrity Number) determined. Fig. 4A and 4B showing agarose gel-electrophoresis and Bioanalyzer gel visualization. The respective RIN numbers and RNA concentrations are presented in Fig. 4C. Sample 1 and 2 showed very low RIN and were not further used. The remaining samples ranged from RIN 4.8 to RIN 7.8. The RNA concentration range was from 50 ng/µl to 418 ng/µl.

### Detection of *spxB* Expression from Plaque Samples

**Figure 5:**
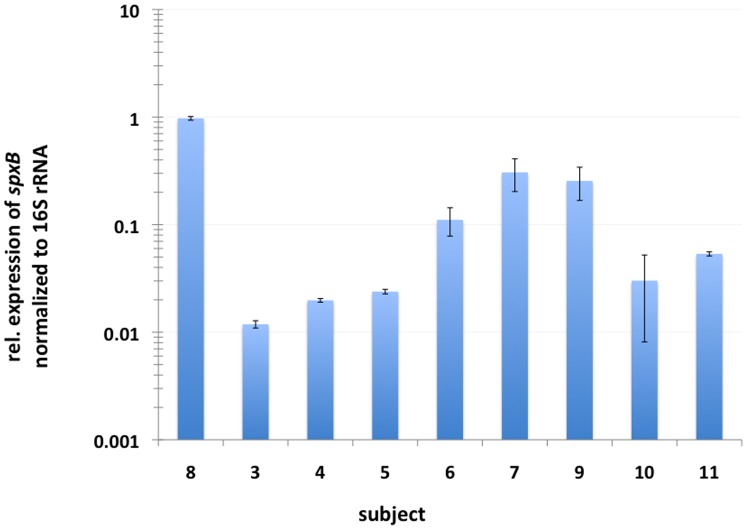
Expression of *spxB* in freshly isolated human oral plaque samples. One time expression measurements of *spxB* from 8 different subjects. Expression was normalized to 16S rRNA expression and subject 8 arbitrary set as 1. Error bars represent standard deviations of technical repeats (n = 3).

Initially the expression of *spxB* in 9 individual plaque samples was compared using 16S rRNA as housekeeping reference. The expression was normalized to the subject with the highest *spxB* expression relative to the others. The result presented in Fig. 5 demonstrates that there is a high degree of interpersonal variation in *spxB* expression. The observed fold difference reached about 88 fold when the highest and the lowest expression were compared (subject 8 vs. subject 3). Contamination by chromosomal DNA was excluded by running a no RT control along with a RT-PCR (data not shown).

**Figure 6:**
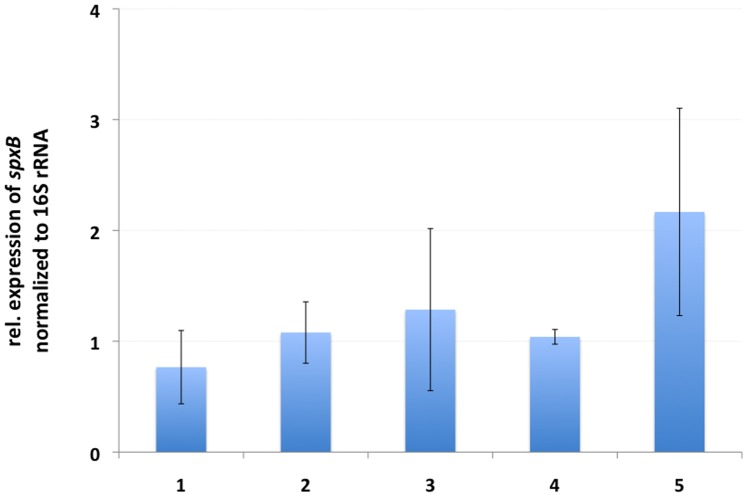
Expression of spxB from one subject over three different days. Time point 1, 2 and 3, 4 were taken on two subsequent days, one hour apart; time point 5 was taken several days later. Expression was normalized to 16S rRNA expression and time point 1 arbitrary set as 1. Error bars represent standard deviations of technical repeats (n = 3).

In addition, the expression of *spxB* from a single subject on five different time-points was determined to learn how stable the expression is on a day-to-day basis. As shown in Fig. 6, time point 1 and 2 as well as 3 and 4 were taken on two subsequent days, one hour apart while time point 5 was taken several days later. Although slight variations are visible, the expression of *spxB* in one subject seems to be constant over time. Furthermore *spxB* expression can be detected repeatable from RNA isolated from human plaque samples.

## Discussion

The HOMD contains DNA sequence information from over 1300 genomes (www.homd.org). An eminent question is what kind of information can be extracted from the deposited sequence data which goes beyond the determination of what is present in the oral cavity or what kind of potential metabolic pathways are encoded [3], [22]. The next logical step is to move away from the static information of sequence data to determine if the microbial potential of the chromosomal sequences is converted to a functional response *in vivo*. The here presented study uses the sequence data provided by HOMD to identify an oral microbial community shared gene, *spxB* and demonstrates its functional expression in freshly isolated human plaque samples.

Several oral commensal *Streptococci* encode the *spxB* gene as we identified in this study. Up to 80% of the detected initial colonizers belong to the genus *Streptococcus*. Some species are even discussed as constant members constituting a core group for initial biofilm formation [23], [24]. The *spxB* gene is therefore highly abundant during initial biofilm formation and might be considered as core gene involved in oral biofilm formation. The production of an antimicrobial substance like H_2_O_2_ could therefore be regarded as an important protection mechanism of the initial colonizers of the resident biofilm community against invading and competing species like the extremely H_2_O_2_ sensitive *S. mutans* [11]. More importantly, it might also be a mechanism to shape the colonization process towards a specific species composition. Only species co-evolved with oral streptococci and therefore adapted to withstand H_2_O_2_ can integrate or colonize in close proximity to the initial colonizers and extend the developing biofilm community [8]. The gene is expressed in the different subjects suggesting a function in the biofilm mode of growth of oral streptococci.

The next step is to design a large-scale study to relate the expression of *spxB* with an assessment of the oral health status. A positive association of *spxB* expression with oral health would allow for a better caries risk assessment in the future. Expression studies on infection relevant genes *in vivo* in humans and animal models have been done before as summarized in [25]. Three *in vivo* studies are relevant in the context of our study: i) An investigation of *in vivo* gene expression in the human host was carried out with *Streptococcus pyogenes* (Group A Streptococcus, GAS) [26]. The author’s demonstrated GAS host-pathogen interactions by analyzing the expression of 17 GAS genes in throat swab specimens sampled from 18 pediatric patients with pharyngitis. Several known and putative virulence genes and regulatory genes were highly expressed during infection [26]; and ii) In a study with the periodontal pathogen *Porphyromonas gingivalis*, Shelbourne *et al.* demonstrated a clear correlation between periodontal disease status and elevated expression of *dnaK* and *htpG* encoding general stress response proteins [27]; iii) while another study established the expression of an uncharacterized gene in *P. gingivalis* (G1334) as more frequent in diseased sites compared to healthy sites [28]. The importance of the G1334 gene in virulence was confirmed in a mouse abscess model of infection [28]. The here presented expression of a community-encoded core gene of oral biofilm formation and the single-species focus by other groups detecting virulence and stress related gene expression *in vivo* demonstrate the feasibility to specifically determine the expression of genes of interest in their ecological context.

Limitations of the study became apparent when the RIN where determined. Although all samples where processed after the same protocol, RNA degradation was a problem for some samples. Two RNA samples were severely degraded and not further considered, while others showed varying degrees of degradation. In general, an RIN of 10 would indicate no degradation. RIN greater than five indicates good total RNA quality for reverse transcription [29]. Some of the samples were close to 5 and therefore would be considered not ideal for *spxB* expression quantification. Nonetheless, we used the total RNA of subject 3 and 4 for cDNA synthesis to determine *spxB* expression. The respective relative expression of *spxB* from subject 3 and 4 were among the lower spectrum, however, subject 5 and 10 showed a similar relative expression level albeit higher RIN. Another caveat of this study lies in the fact that the oral microbial diversity among subjects varies [30] and the *spxB* sequences will have sequence dissimilarities. This will result in nucleotide mis-pairing of the *spxB* oligonucleotides used for *spxB* expression. As a consequence, non-ideal PCR amplification conditions will occur due to different primer efficiencies when plaque samples with undefined species composition are used. This might be prevented in the future if more *spxB* sequences become available to optimize oligonucleotides for amplification. In addition, the main advantage we take in our approach is the wide distribution of a highly conserved gene among the most prominent genus in the oral biofilm. If other similar important genes can be identified needs to be determined.

In summary, we showed *in vivo* expression of an oral biofilm community encoded gene in its ecological context, thus suggesting an active role of *spxB* in oral biofilm physiology. This study demonstrates how the HOMD database can be used to determine a dynamic relationship or the functionality of the potential encoded in the metagenome of the oral biofilm.
